# The Forward Testing Effect Is Resistant to Acute Psychosocial Retrieval Stress

**DOI:** 10.1027/1618-3169/a000571

**Published:** 2023-03-14

**Authors:** Bernhard Pastötter, Bernadette von Dawans, Gregor Domes, Christian Frings

**Affiliations:** ^1^ Department of Cognitive Psychology, University of Trier, Germany; ^2^ Department of Biological and Clinical Psychology, University of Trier, Germany

**Keywords:** episodic memory, testing effects, acute psychosocial stress, cortisol, Trier Social Stress Test for Groups (TSST-G)

## Abstract

**Abstract:** The forward testing effect refers to the finding that testing of previously studied information improves memory for subsequently studied newer information. Recent research showed that the effect is immune to acute psychosocial encoding/retrieval stress, i.e., stress that is induced before initial encoding. The present study investigated whether the forward testing effect is also robust to acute psychosocial retrieval stress, i.e., stress that is induced after encoding but before retrieval of the critical item list. Participants (*N* = 128) studied three lists of words in anticipation of a final cumulative recall test. Participants were tested immediately on Lists 1 and 2 (testing condition) or restudied the two lists after initial study (restudy condition). After study of the critical List 3, psychosocial stress was induced in half of the participants (stress group), whereas no stress was induced in the other half (control group). The Trier Social Stress Test for Groups (TSST-G) was used for stress induction. Salivary cortisol, alpha amylase, and subjective stress were repeatedly measured. The results of the criterion test showed a generally detrimental effect of psychosocial retrieval stress on List 3 recall. Importantly, the forward testing effect was unaffected by retrieval stress. The findings are discussed with respect to current theories of the forward testing effect.


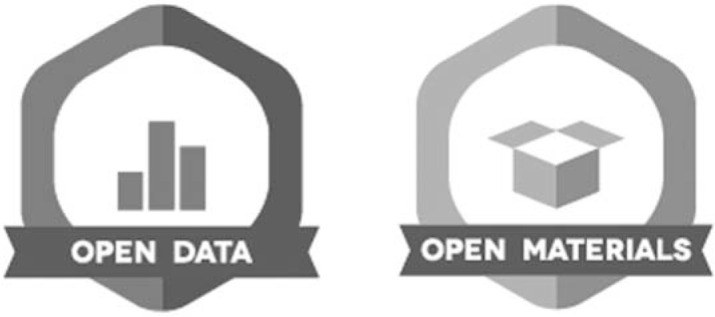


Acute stress can have profound effects on human memory (for a review, see Vogel & Schwabe, 2016). For instance, regarding long-term memory, acute social and/or physical stress is often found to impair episodic memory retrieval (e.g., Kuhlmann et al., 2005; Schwabe & Wolf, 2014), whereas semantic memory retrieval seems to benefit from acute stress (Smith, Hughes, et al., 2019). Smith and Thomas (2018) reviewed potential interventions that could reduce the negative effect of acute stress on episodic memory retrieval and argued that retrieval practice, i.e., the act of retrieving information from long-term memory, could be an effective intervention in itself.

Retrieval practice can have several positive effects, two of them being the backward and forward testing effects. The backward testing effect refers to the well-established finding that retrieval practice of previously studied information enhances its long-term retention more than other form or reprocessing (e.g., restudy) do (for a review, see Karpicke, 2017). The forward testing effect, on the other hand, describes that retrieval practice potentiates new learning and enhances memory for subsequently studied other information. Many studies have shown that the forward testing effect is a very robust phenomenon that can be reliably observed for different materials (e.g., words, pictures, videos) and populations (for reviews, see Pastötter & Bäuml, 2014; Yang et al., 2018).

Both the backward and forward testing effects have been examined in stress research. Smith et al. (2016) examined the backward effect with regard to retrieval stress, i.e., stress that is induced prior to the criterion test. On the first day, participants studied a list of items (words and pictures), half of which were subsequently restudied and the other half tested, i.e., retrieval practiced. On the next day, half of the participants were stressed with the Trier Social Stress Test for Groups (TSST-G) protocol (von Dawans et al., 2011), i.e., a group format of the standard TSST protocol, while the other half were not. In a subsequent memory test, the stressed participants remembered relatively fewer restudied items from Day 1 than the nonstressed participants. In contrast, no difference between groups was found for the retrieval practiced items. Thus, retrieval practice of the tested items on Day 1 protected these items against the negative effect consequences of retrieval stress at Day 2. In addition, retrieval practice in comparison to restudy of items on Day 1 was found to reduce the post-stress false alarm rate in a recognition test on Day 2 (Smith, Race, et al., 2019).

Pastötter et al. (2020) examined the forward testing effect with regard to encoding/retrieval stress, i.e., stress that is induced before the encoding (and retrieval) of item material within a single session. Acute psychosocial stress was induced in half of the participants with the TSST-G protocol before initial encoding of three unrelated word lists. Participants were tested immediately on Lists 1 and 2 in the testing condition or restudied the two lists after initial study in the restudy condition. Next, participants studied and were tested on List 3. The results showed that testing in comparison to restudy between lists enhanced correct recall of List 3 and also reduced the number of prior list intrusions. Importantly, the forward testing effect was equally present in the stress group and the control group. Encoding/retrieval stress had no significant main effect on List 3 recall, which is consistent with the results reported in a meta-analysis by Shields et al. (2017). Relatedly, Szőllősi et al. (2017) examined the effect of acute retrieval stress on the test-potentiated learning effect, i.e., the indirect benefit of retrieval practice on subsequent relearning of the practiced information, and also found no stress effect.

The forward testing effect is a multi-mechanism phenomenon, and both encoding and retrieval explanations of the effect have been suggested (see Yang et al., 2018). Encoding explanations assume that testing between lists resets encoding processes, thus increasing attention and reducing memory load during encoding (Pastötter et al., 2011, 2018), or induces participants to switch to more effective (e.g., more elaborative) encoding strategies (Chan et al., 2018). Retrieval explanations assume that testing between lists drives mental context change, which enhances list differentiation and reduces (proactive) interference at test (Bäuml & Kliegl, 2013). Importantly, different processes appear to contribute to the forward testing effect for different materials. Kliegl and Bäuml (2021, 2022) provided evidence that reset of encoding contributes to the effect for both (semantically) related and unrelated word lists, whereas encoding strategy change contributes mainly for related lists and proactive interference reduction contributes mainly for unrelated lists.

Pastötter et al. (2020) used unrelated word lists as item material. Therefore, it could be argued that neither encoding nor retrieval processes, i.e., neither reset of encoding nor proactive interference reduction, were affected by acute psychosocial stress. However, this conclusion might be premature. Indeed, it could also be argued that one process, e.g., reset of encoding, was enhanced by stress, whereas the other process, e.g., proactive interference reduction, was impaired by stress, thereby canceling out any overall effect of stress in the Pastötter et al. (2020) study. In fact, this very argument was also made in the Shields et al. (2017) meta-analysis on stress effects to explain the general lack of an encoding/retrieval stress effect on episodic memory. Thus, it needs to be shown to what extent one of the two processes is affected by acute retrieval stress independently of the other, i.e., in isolation. In a single-session experiment using unrelated item material, this can be well accomplished by inducing retrieval stress in participants after encoding but before retrieval of the target list – this should affect solely retrieval but not encoding processes of the forward testing effect. The present experiment did exactly this. The results of the present experiment will allow us to draw firm conclusions about the specific influences of acute psychosocial stress on reset of encoding and proactive interference reduction, i.e., two central theories of the forward testing effect, conclusions that could not be drawn from the Pastötter et al. (2020) study alone.

Participants studied three unrelated word lists. List 3 was the critical list. In the testing condition, participants were tested on Lists 1 and 2 after initial study, whereas in the restudy condition, they restudied the two lists. Acute psychosocial stress was induced in the stress group, using the TSST-G protocol (von Dawans et al., 2011) after encoding and before retrieval of List 3. Thus, acute stress could affect only retrieval (i.e., proactive interference reduction) but not encoding processes (i.e., reset of encoding) of the forward testing effect. No stress was induced in the control group. If acute stress modulates proactive interference reduction, we should observe a modulation of the forward testing effect by retrieval stress, i.e., an interaction between condition and group for List 3 recall. Such finding could be attributed solely to proactive interference reduction and not to reset of encoding. Thus, the present research question is particularly interesting from a theoretical point of view in terms of two prominent explanations for the forward testing effect, i.e., reset of encoding and proactive interference reduction.

## Method

### Participants

Sample size was calculated a priori using G*Power (version 3.1.9.4; Faul et al., 2007). Required sample size was calculated for a medium interaction effect in a 2 × 2 fixed-effects analysis of variance (ANOVA) with the between-subjects factors of condition (testing, restudy) and group (stress, control). Regarding input parameters, α was set to .05, 1 − β was set to .80, and the size of the interaction effect of interest was set to *f* = .25 (medium). Based on these input variables, G*Power suggested a total sample size of 128 subjects, i.e., 32 participants per condition and group.

One hundred and twenty-eight students from the University of Trier participated in the study (mean age: 22.0 years, *SD* = 2.9 years; 104 females, 24 males) and were quasi-randomly assigned to conditions and groups with equal distribution of females and males (26 females and 6 males in each combination of condition and group). Participants’ age did not differ significantly between conditions and groups, Fs<1. Usage of hormonal contraceptives in women was equally distributed across stress and control groups, $\chi^{2}\left(1\right)<1$. Note that the question on hormonal contraceptives was asked to only 50 of the 104 women because the item on hormonal contraceptives was not included in the online questionnaire until the middle of the data collection phase.

Exclusion criteria were acute or chronic illness including neurological disorders, psychiatric disorders within lifetime, substance abuse within the last 6 months, and medication intake (except oral contraceptives). Data for these criteria were obtained from interested subjects via an online questionnaire before participation. Subjects received either course credit or 20 Euro for participation.

### Material

For the memory task, the same item material as in the study by Pastötter et al. (2020) was used. The material was taken from Pastötter et al. (2012; Experiment 2), in which 144 German nouns of medium frequency and word length of 4–8 letters were used. For each participant, 12 nouns were randomly assigned to each of the three lists. Different words were used for each list.

### Design

**Figure 1:**
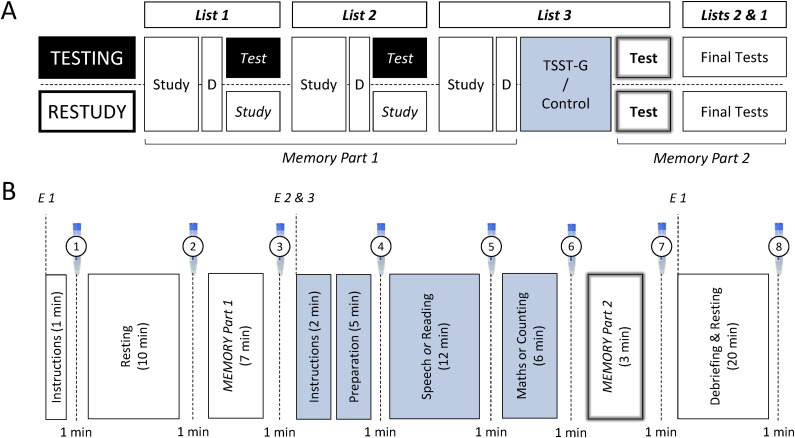
Procedure. (A) Memory task. In both the testing and the restudy condition, participants studied three lists of items, each followed by a short distractor task. List 3 was the critical list. D = distractor; TSST-G = Trier Social Stress Test for Groups. (B) Phases of the experiment. There were 8 time points of combined saliva collection and subjective stress rating. E = Experimenter.

The experiment had a 2 × 2 design with the between-subjects factors of practice strategy (testing, restudy) and stress induction (stress, control). In the testing condition, Lists 1 and 2 were tested after initial study of each of the two lists (and a short distractor), whereas in the restudy condition, Lists 1 and 2 were restudied after initial study (and a short distractor; see Figure 1A). The TSST-G protocol was administered comprising a stress and a control group (von Dawans et al., 2011).

### Procedure

All data were collected in the afternoon to minimize the impact of diurnal variations in cortisol secretion. The single sessions started between 2 and 6 p.m. Participants were invited to the laboratory in small groups of 2–4 participants (the number of participants per session was not counterbalanced between conditions and groups and therefore was considered as covariate in all statistical analyses). They were informed in advance that they should not smoke 3 h before the experimental session. The assignment of participants to stress and control groups was quasi random with the constraint that males and females were equally distributed across groups. Upon arrival at the laboratory, participants were instructed not to communicate with each other at any time throughout the experiment. Participants were seated at computers in individual cubicles and asked to read and sign the informed consent. Mobile walls were used to restrict eye contact and social interaction between participants. Participants were allowed to drink at specified times during the experiment, but not 10 min before saliva sampling.

#### Memory Task

Participants took part in either the testing condition or the restudy condition. In both conditions, participants studied three 12-item lists (see Figure 1A). Items of each list were visually presented in random order in the middle of a computer screen with a presentation rate of 5 s (4.5 s of item presentation, 0.5 s of blank screen). Each participant was sitting in front of their own computer and monitor. After the presentation of each list (1 min), participants solved simple mathematics (e.g., 4×8) for 30 s as a distractor. Conditions differed in interlist activity that followed the distractors after Lists 1 and 2. In the testing condition, after study of List 1 and the distractor, participants were given 1 min to recall in any order they wished as many items as they could remember from List 1; next, after study of List 2 and the distractor, participants were given 1 min to recall in any order they wished as many items as they could remember from List 2. In contrast, in the restudy condition, participants were re-presented the just-studied items of Lists 1 and 2 (1 min each) with new random item presentation order. Then, in both conditions, participants studied List 3, did the distractor task (30 s), and were tested on List 3. In the immediate List 3 recall test, participants were given 1 min to recall in any order they wished as many items as possible from List 3. After this, Lists 2 and 1 were tested in two final recall tests (1 min each); List 2 was always tested before List 1 (as in Pastötter et al., 2020). In all recall tests, participants typed in responses on a computer keyboard.

#### TSST-G Protocol

The TSST-G protocol (von Dawans et al., 2011) was used for stress induction between study of List 3 (and the short distractor task) and the recall test of List 3. Acute psychosocial stress was induced in half of the participants (stress group) before the criterion test of List 3. No stress was induced in the other half of the participants (control group). The stress induction phase between study and test of List 3 lasted about 28 min. Eight saliva samples (Salivette; Sarstedt, Nümbrecht, Germany) were collected from each participant throughout the session. Exact time points of saliva collection are depicted in Figure 1B. The detailed TSST-G protocol, including saliva collection and subjective stress ratings, and the data analysis are described in the Electronic Supplemental Materials (ESM 1).

### Data Analysis

Both frequentist and Bayesian statistics were calculated with JASP 0.16.3 (JASP Team, 2022). Percent correct list recall and number of prior list intrusions were examined as dependent variables in analyses of covariance (ANCOVAs) with number of participants per session (2–4) as a covariate in all analyses. Regarding Bayes statistics, ${BF}_{01}$ is reported when the Bayesian analysis provides relatively more evidence for the null hypothesis than for the alternative hypothesis; otherwise, ${BF}_{10}$ is reported. Standard priors were used for the calculation of Bayes factors (*r* scale fixed effects: 0.5; *r* scale random effects: 1; *r* scale covariates: 0.354). To determine the strength of evidence, we used Jeffreys’s (1998) benchmarks, with Bayes factors corresponding to anecdotal (0–3), substantial (3–10), strong (10–30), very strong (30–100) or decisive (>100) evidence in favor of the null (${BF}_{01}$) or alternative hypothesis (${BF}_{10}$).

## Results

### Manipulation Checks

All manipulation checks regarding physiological responses (cortisol, alpha amylase) and subjective stress ratings (subjective stress experience, mood, physical well-being, physical tension, feeling of control, need for contact) were successful, thus validating the stress induction (see ESM 1 for the detailed methods, descriptive statistics, and statistical results).

### Immediate Recall of Lists 1 and 2

The results of the immediate recall tests in the testing condition are shown in Table 1. A 2 × 2 repeated-measures ANOVA with the factors of list (List 1 vs. List 2) and stress induction (stress vs. control), and number of participants per session as a covariate, showed no significant main effects of list, $F\left(1,\;61\right)=1.76,\;MSE=168.11,\;p=.189{(BF}_{01}=2.10$, anecdotal evidence, compared to null model), or practice strategy, $F\left(1,\;61\right)<1{(BF}_{01}=3.09$, substantial evidence, compared to null model), and no significant interaction between factors, $F\left(1,\;61\right)=1.16,MSE=168.11,\;p=.285{\;(BF}_{01}=2.91$, anecdotal evidence, compared to two main-effects model).

**Table 1:** Immediate and final recall results as a function of condition (practice strategy) and group (stress induction)

Group	Test	Condition	Recall rates (%)			Intrusions (no)
			List 1	List 2	List 3	List 3
Stress	Immediate recall	Testing	72.14 (2.14)	66.67 (3.46)	39.58 (4.63)	1.22 (0.38)
		Restudy			28.39 (5.20)	2.31 (0.46)
	Final recall	Testing	46.35 (4.05)	41.41 (5.31)		
		Restudy	56.77 (5.15)	49.74 (5.53)		
Control	Immediate recall	Testing	72.40 (3.07)	71.09 (2.92)	50.78 (3.88)	0.59 (0.33)
		Restudy			35.16 (5.65)	2.63 (0.66)
	Final recall	Testing	40.63 (3.99)	50.52 (4.41)		
		Restudy	61.20 (4.67)	47.92 (5.74)		
Means and *SEs* of the mean (in parentheses).						

### Final Recall of List 3

The results of the critical List 3 recall test are presented in Table 1. Regarding correct recall, a 2 × 2 ANCOVA with the between-subject factors of practice strategy (testing vs. restudy) and stress induction (stress vs. control), and number of participants per session as a covariate, revealed significant main effects of practice strategy, $F\left(1,\;123\right)=5.95,\;MSE=757.89,\;p=.016,\;\eta_{p}^{2}=.046\;({BF}_{10}=3.26$, substantial evidence, compared to null model), and stress induction, $F\left(1,\;123\right)=4.23,MSE=757.89,\;p=.042,\;\eta_{p}^{2}=.033\;({BF}_{10}=1.36$, anecdotal evidence, compared to null model). The interaction between the two factors was not significant, *F*(1, 123)<1 $({BF}_{01}=4.11$, substantial evidence, compared to two main-effects model). These results suggest a reliable positive FTE on List 3 recall that was not moderated by stress. In addition, stress generally had a negative influence on correct List 3 recall.

Regarding prior list intrusions, a 2 × 2 ANCOVA showed a significant main effect of practice strategy, $F\left(1,\;123\right)=9.94,MSE=7.22,\;p=.002,\;\eta_{p}^{2}=.075\;({BF}_{10}=17.25$, strong evidence, compared to null model), but neither a significant main effect of stress induction, F(1, 123)<1 $({BF}_{01}=4.77$, substantial evidence, compared to null model), nor a significant interaction, F(1, 123)<1 $({BF}_{01}=3.08$, substantial evidence, compared to two main-effects model). These results suggest a reliable positive FTE on prior list intrusions that was unaffected by stress.

### Final Recall of Lists 2 and 1

The results of the final recall tests are shown in Table 1. Two 2 × 2 ANCOVAs with the factors of practice strategy (testing vs. restudy) and stress induction (stress vs. control), and number of participants per session as a covariate, were calculated separately for List 2 and List 1. Regarding List 2, the ANCOVA showed no significant main effects of practice strategy, $F\left(1,\;123\right)<1{\;(BF}_{01}=4.28$, substantial evidence, compared to null model), or stress induction, $F\left(1,\;123\right)<1{\;(BF}_{01}=3.76$, substantial evidence, compared to null model), and no significant interaction, $F\left(1,\;123\right)<1\;({BF}_{01}=2.83$, anecdotal evidence, compared to two main-effects model). Regarding List 1, the ANCOVA revealed a significant main effect of practice strategy, $F\left(1,\;123\right)=12.14,MSE=648.46,\;p<.001,\;\eta_{p}^{2}=.090{\;(BF}_{10}=40.60$, very strong evidence, compared to null model), but no significant main effect of stress induction, $F\left(1,\;123\right)<1{\;(BF}_{01}=5.36$, substantial evidence, compared to null model), and no significant interaction, $F\left(1,\;123\right)=1.38,\;MSE=648.46,\;p=.243{\;(BF}_{01}=2.40$, anecdotal evidence, compared to two main-effects model).

## Discussion

In the present study, we set out to examine whether acute psychosocial retrieval stress influences the forward testing effect. Manipulation checks regarding both physiological and subjective stress measures were successful. The results showed a significant forward testing effect, with interim testing in comparison to restudy enhancing correct recall and reducing the number of prior list intrusions in the List 3 recall test. Importantly, the forward testing effect was found independent of retrieval stress for both correct recall and prior list intrusions. Together with the results of a recent study by Yang et al. (2020), these results suggest that neither psychosocial stress nor test anxiety moderates the forward testing effect. Moreover, in the present study, a generally detrimental effect of retrieval stress on List 3 recall was observed, which is consistent with previous stress research (e.g., Kuhlmann et al., 2005; Schwabe & Wolf, 2014). Together with the findings of Pastötter et al. (2020), these results suggest that neither encoding (i.e., reset of encoding) nor retrieval processes (i.e., proactive interference reduction) of the forward testing effect are influenced by acute stress.

Different processes may contribute to the forward testing effect for different materials. In particular, Kliegl and Bäuml (2021, 2022) argued that reset of encoding and proactive interference reduction contribute mainly to the effect for unrelated item lists, whereas reset of encoding and encoding strategy change contribute mainly to the forward effect for semantically related item material. In contrast to the retrieval from episodic memory, which is typically impaired under retrieval stress, the processing and retrieval of semantic memories has been shown to benefit from acute psychosocial stress (Smith, Hughes, et al., 2019). Based on this recent research, it could be hypothesized that acute psychosocial stress, induced either before encoding or retrieval of the episodic memory task, reduces or even eliminates the forward testing effect for semantically related item materials. Indeed, acute stress should promote elaborative encoding and/or retrieval of the items in both the testing and restudy conditions, which should reduce or even eliminate any strategy change benefits due to retrieval practice in the testing condition. Such finding would have both theoretical and practical implications, and thus, it is a high priority to investigate this issue in future research.

Smith et al. (2016) examined the influence of retrieval stress on the backward testing effect when there was a one-day delay between retrieval practice and the final test. They found that retrieval practice eliminated the negative stress effect that was observed for the restudied items. In contrast to Smith et al. (2016), the present study was a single-session experiment with a relatively short retention interval between retrieval practice and final test. With such a short retention interval, the backward testing effect is typically eliminated or even reversed (e.g., Roediger & Karpicke, 2006; Toppino & Cohen, 2009). A reversal of the backward testing effect was also observed in the present final recall test of List 1. Importantly, no influence of retrieval stress on the reversal was observed, which suggests that the restudy advantage after short retention intervals is robust to retrieval stress. Together with the findings of Smith et al. (2016), these results suggest that the backward testing effect (after longer delay) is affected by retrieval stress (i.e., the effect is larger following stress), but the reversed testing effect (after shorter delay) may be not. However, note that List 1 recall rates should be considered only as a rough measure of the (reversed) backward testing effect as final List 1 recall may have been influenced at test by output interference from the preceding List 3 and List 2 recall tests (see Yang et al., 2022). Thus, future research may like to re-examine this issue with a purer measure of the (reversed) backward testing effect after short retention interval.

To conclude, the results of this study indicate that the forward testing effect is robust to acute psychosocial retrieval stress. Together with the findings from the previous forward testing effect study by Pastötter et al. (2020), the results suggest that neither reset of encoding nor proactive interference reduction is affected by acute psychosocial (encoding or retrieval) stress. Because there were relatively few men in the present sample, future research may like to address the generalizability of the results. Future research is also needed to examine the interaction between stress and testing effects for more related materials and more naturalistic settings – and also with stressors that are more related to the content of the studied material. The results from the basic experimental research so far are promising that retrieval practice is a very effective learning strategy that can be used in stressful situations.

## Electronic Supplementary Material

The electronic supplementary material is available with the online version of the article at https://doi.org/10.1027/1618-3169/a000571

**ESM 1.** The electronic supplementary material describes the detailed TSST-G protocol and reports the data analyses and results regarding physiological stress responses (cortisol, alpha amylase) and subjective stress ratings (subjective stress experience, mood, physical well-being, physical tension, feeling of control, need for contact).

## Notes

**Publication Ethics:** The study was carried out in accordance with the recommendations of the Declaration of Helsinki. Both the memory task and the TSST-G protocol were approved by the local ethical review committee at the University of Trier. All participants gave written informed consent before participation.

**Authorship:** Bernhard Pastötter, development of study concept and experimental design – data analysis – data curation – original draft, writing – review & editing; Bernadette von Dawans, review & editing; Gregor Domes, review & editing; Christian Frings, review & editing. All authors approved the final version of the article.

**Open Data:** All data and analysis codes can be downloaded at Open Science Framework: https://osf.io/ex4ru/ (Pastötter et al., 2022).
