# Supplementary material for: The Forward Testing Effect Is Resistant to Acute Psychosocial Retrieval Stress
Source: Exp Psychol. 2023 Mar 14;70(1):32–9. doi: 10.1027/1618-3169/a000571 (PMC10388236; doi:10.1027/1618-3169/a000571)
Supplement: Supplementary file 1 [file zea_70_1_32_esm1.pdf]

https://doi.org/10.1027/1618-3169/a000571

Electronic Supplemental Materials (ESM)

The forward testing effect is resistant to acute psychosocial retrieval stress

Bernhard Pastötter[1], Bernadette von Dawans[2], Gregor Domes[2], Christian Frings[1]

[1]Department of Cognitive Psychology, University of Trier, Germany

[2]Department of Biological and Clinical Psychology, University of Trier, Germany

Corresponding author:

Bernhard Pastötter (pastoetter@uni-trier.de)

Method

TSST-G protocol

Stress Condition

The TSST-G protocol was administered (von Dawans et al., 2011, 2012). Data from 73 participants were collected in 2019, i.e., before the Corona pandemic, and data from 55 more participants were collected in 2021. No data were collected in 2020. No face masks were worn by participants or experimenters during the 2019 sessions. In contrast, in all 2021 sessions, all participants and experimenters wore face masks throughout the experiment. In addition, in 2021 but not in 2019, all work surfaces, saliva testing utensils, keyboards, and monitors were disinfected after each session. Because these factors were not experimentally manipulated, they were not considered further in the present analyses. For the interested reader, these data are stored on the OSF, https://osf.io/ex4ru/.

In the stress condition, after study of list 3 (and the short distractor task), participants were instructed by the experimenter to prepare a speech for a mock job interview (2 min). Participants were given 5 min to prepare the application and outline a short speech. They were not allowed to use any notes during the interview. Participants were told that the interview would be in front of two jury members who were experts in nonverbal behavior and were videotaped by one camera. The experimenter pretended that the interviews would be video-recorded for later analysis of participants' verbal and nonverbal behavior (in fact, all videos were deleted at the end of the session for data-protection

reasons). The video camera was standing conspicuously in the middle of the room next to the jury members' table.

After the preparation phase, the experimenter left, and two female jury members entered the room. They wore white laboratory coats. They were trained to withhold verbal and nonverbal feedback. While the jury members were sitting behind their table in the middle of the room, participants were instructed to stand in their cubicles and look at the jury. Participants were called on in random order to start their speech. Depending on the number of participants in a session (2 to 4), different speech presentation times were assigned to participants. In groups of 2 participants, each participant was instructed to give a 6 min speech; in groups of 3, a 4 min speech, and in groups of 4, a 3 min speech. Thus, in all groups, all speeches together lasted 12 min. Whenever a participant finished speaking before the allotted time expired, the committee responded in a standardized way by first pausing for 20 seconds, then pointing out that there was still time left and the participant should continue. If the participant stopped a second time the jury asked prepared standard questions according to the TSST protocol (Kirschbaum et al., 1993).

After all participants have given their speech, participants in the stress condition did a difficult backward counting task while standing in their cubicles and looking to the jury and the camera (see (see von Dawans et al., 2012). Participants serially subtracted the number 17 from a given four-digit number aloud and as quickly and accurately as possible (e.g., 4878, 4862, etc.). Participants were called on in random order to start their calculation. If participants made a mistake, they had to restart at their personal number. Participants were interrupted and called upon several times, resulting in a total of about 3 min of calculating for each participant in groups of 2 participants, 2 min in groups of 3, and 1.5 min in groups of 4. After the 6 min calculation phase, the test phase of the memory task started, and participants were asked to recall the item of the target list 3. Thus, the test phase of the memory task began about 28 min after stressor onset, i.e., the instruction to prepare the speech.

Control Condition

In the control condition, after study of list 3, participants were given a popular scientific text that they should prepare for low voice reading in self-paced reading speed. Participants were explicitly told that their reading performance would not be evaluated. After the preparation phase, the experimenter left and two female jury members (the same as in the stress condition) entered the room and sat down behind the table in the middle of the room. The jury members wore casual clothes. No video camera was present in the room. Participants were asked to read out the scientific text simultaneously in a

low voice for 12 min. Participants were neither evaluated nor interrupted. After the reading phase, participants were instructed to enumerate series of numbers in increments of 2, 5, 10 or 20 simultaneously in a low voice for 6 min (e.g., 2, 4, 6, 8, 10, etc.). Participants were explicitly told that their counting performance would not be evaluated.

Saliva Collection and Subjective Ratings

Eight saliva samples (Salivette; Sarstedt, Nümbrecht, Germany) were collected from each participant throughout the session. Each saliva collection took 1 min and was accompanied by a subjective rating of the current stress level ("not at all stressed" to "very much stressed"), current mood ("very bad" to "very good"), physical wellbeing ("very well" to "very unwell"), physical tension ("no tension at all" to "very high tension"), feeling of control ("very low" to "very high"), and the wish to contact a familiar person ("no need at all" to "very high need") on 11-point scales ranging from 0 to 100 (with 10-point increments). All ratings were forced choice; each response was directly followed by the next display.

Data Analysis

After completion of a session, the saliva tubes were stored at -20° C until biochemical analysis at the University laboratory. Both salivary cortisol and alpha-amylase responses were analyzed. For cortisol analysis, a time-resolved fluorescence immunoassay was used (Dressendörfer et al., 1992); 100 µl (50 µl per well) of saliva were used for duplicate analysis. For alpha-amylase analysis, the chromogenic molecule 2-Chloro-4-nitrophenyl-a-D-maltotrioside was used as the substrate (Lorentz et al., 1999); saliva was diluted 1:200 with assay diluent and 16 µl (8 µl per well) of the diluted saliva were used for duplicate analysis. For both analyses, the intra- and inter-assay coefficients of variation were below 10%.

For manipulation checks regarding stress induction, salivary cortisol (in nmol/l), alpha amylase (in U/l), and subjective ratings were examined in separate repeated-measures ANOVAs with the between-subjects factors stress induction (stress, control) and the within-subjects factor time point of measurement (1 to 8). Number of participants per session (2 to 4) was included as a covariate in all ANOVAs. Greenhouse-Geisser correction was applied where necessary. Holm-Bonferroni corrected multiple comparisons between groups were calculated for time points of measurement 4 to 8, for which stress effects could be expected. One participant in the stress group did not follow instructions regarding saliva sampling and therefore did not enter analysis of salivary cortisol and alpha amylase.

One other participant in the stress group did not enter analysis of alpha amylase due to missing values from the biochemical analysis. Both frequentist and Bayes statistics were calculated.

Results

Cortisol

Means and standard deviations are shown in Table S1. A 2 × 8 repeated-measures ANOVA with the between-subjects factor of stress induction (stress vs. control) and the within-subjects factor of time point of measurement (1 to 8), and number of participants per session as a covariate, showed a significant main effect of stress induction, $F(1, 124) = 10.43, MSE = 44.87, p = .002, \eta_p^2 = .078$ ($BF_{10} = 22.13$, strong evidence, compared to null model), and a significant interaction between factors, $F(1.62, 201.45) = 12.35, MSE = 15.92, p < .001, \eta_p^2 = .091$ ($BF_{10} > 100$, decisive evidence, compared to two-main-effects model). The main effect of time of measurement was not significant in null-hypothesis significance testing, $F(1.62, 201.45) = 1.16, MSE = 15.92, p = .308$; however, Bayes statistics indicated decisive evidence for such effect ($BF_{10} > 100$). Regarding the interaction, Holm-Bonferroni corrected pairwise comparisons indicated that cortisol levels were significantly increased in the stress group compared to the control group at time points 5 to 8, $ps < .05$. Thus, stress induction was successful regarding cortisol. Correlational analysis of individual cortisol, averaged over time points 5 to 8, and individual list 3 recall performance showed no significant (Pearson's) partial correlations (number of participants per session partialed out), overall: $r = -0.120, p = .182$; stress group: $r = -0.116, p = .368$; control group: $r = 0.032, p = .803$.

Alpha Amylase

Means and standard deviations are shown in Table S1. A 2 × 8 repeated-measures ANOVA with the factors of stress induction (stress vs. control) and time point of measurement (1 to 8), and number of participants per session as a covariate, revealed a significant main effect of time point of measurement, $F(3.15, 387.12) = 7.45, MSE = 5965.08, p < .001, \eta_p^2 = .057$ ($BF_{10} > 100$, decisive evidence, compared to null model), and a significant interaction between the two factors, $F(3.15, 387.12) = 8.62, MSE = 5965.08, p < .001, \eta_p^2 = .065$ ($BF_{10} > 100$, decisive evidence, compared to two-main-effects model). The main effect of stress induction was not significant, $F(1, 123) = 2.55, MSE = 101403.73, p = .113$ ($BF_{01} = 1.04$, anecdotal evidence, compared to null model). Holm-Bonferroni corrected pairwise comparisons showed that alpha amylase level was significantly increased in the stress group compared to the control group at time point 5, $p < .05$. Thus, stress induction was successful regarding alpha amylase. Correlational analysis of individual

alpha amylase at time point 5 and individual list 3 recall performance revealed no significant (Pearson's) partial correlations (number of participants per session partialed out), overall: $r = -0.025, p = .785$; stress group: $r = -0.095, p = .464$; control group: $r = -0.091, p = .477$.

Subjective Ratings: Stress

Subjective stress experience was rated from 0 ("not at all stressed") to 100 ("very much stressed"). Means and standard deviations of the subjective stress ratings are shown in Table S2. A 2 × 8 repeated-measures ANOVA with the factors of stress induction (stress vs. control) and time point of measurement (1 to 8), and number of participants per session as a covariate, showed a significant main effect of stress induction, $F(1,125) = 18.71, MSE = 2044.80, p < .001, \eta_p^2 = .130$ ($BF_{10} > 100$, decisive evidence, compared to null model), a significant main effect of time point of measurement, $F(4.19, 523.99) = 7.47, MSE = 454.45, p < .001, \eta_p^2 = .056$ ($BF_{10} > 100$, decisive evidence, compared to null model), and a significant interaction between the two factors, $F(4.19, 523.99) = 8.49, MSE = 454.45, p < .001, \eta_p^2 = .064$ ($BF_{10} > 100$, decisive evidence, compared to two-main-effects model). Regarding the interaction, Holm-Bonferroni corrected pairwise comparisons revealed that the subjective stress was rated significantly higher in the stress group than in the control group at all time points 4 to 8, all $ps < .05$.

Subjective Ratings: Mood

Current mood was rated from 0 ("very bad") to 100 ("very good"). Means and standard deviations of the subjective mood ratings are shown in Table S2. A 2 × 8 repeated-measures ANOVA with the factors of stress induction (stress vs. control) and time point of measurement (1 to 8), and number of participants per session as a covariate, revealed a significant main effect of stress induction, $F(1, 125) = 8.38, MSE = 1403.27, p = .005, \eta_p^2 = .063$ ($BF_{10} = 7.28$, substantial evidence, compared to null model), and a significant interaction between the two factors, $F(5.08, 634.51) = 6.02, MSE = 223.70, p < .001, \eta_p^2 = .046$ ($BF_{10} > 100$, decisive evidence, compared to two-main-effects model). The main effect of time of measurement was not significant in null-hypothesis significance testing, $F(5.08, 634.51) = 1.42, MSE = 223.70, p = .213$, whereas Bayes analysis provided decisive evidence for such effect ($BF_{10} > 100$). Regarding the interaction, Holm-Bonferroni corrected pairwise comparisons suggested that subjective mood was rated as significantly worse in the stress group than in the control group at all time points 4 to 8, all $ps < .05$.

Subjective Ratings: Physical Wellbeing

Physical wellbeing was rated from 0 (“very well”) to 100 (“very unwell”). Means and standard deviations of the ratings are presented in Table S2. A 2 × 8 repeated-measures ANOVA with the factors of stress induction (stress vs. control) and time point of measurement (1 to 8), and number of participants per session as a covariate, showed a significant main effect of time point of measurement, $F(5.00, 624.57) = 5.38, MSE = 273.16, p < .001, \eta_p^2 = .041$ ($BF_{10} > 100$, decisive evidence, compared to null model), and a significant interaction between the two factors, $F(5.00, 624.57) = 2.92, MSE = 273.16, p = .013, \eta_p^2 = .023$ ($BF_{10} = 4.93$, substantial evidence, compared to two-main-effects model). The main effect of stress induction was not significant, $F(1,125) = 2.34, MSE = 2569.11, p = .128$ ($BF_{01} = 1.62$, anecdotal evidence, compared to null model). Holm-Bonferroni corrected pairwise comparisons showed that physical wellbeing was significantly lower in the stress group than in the control group at time point 4, $p < .05$.

Subjective Ratings: Physical Tension

Experience of physical tension was rated from 0 (“no tension at all”) to 100 (“very high tension”). Means and standard deviations of the ratings are shown in Table S2. A 2 × 8 repeated-measures ANOVA with the factors of stress induction (stress vs. control) and time point of measurement (1 to 8), and number of participants per session as a covariate, revealed a significant main effect of stress induction, $F(1,125) = 10.97, MSE = 2372.61, p = .001, \eta_p^2 = .081$ ($BF_{10} = 22.50$, strong evidence, compared to null model), a significant main effect of time point of measurement, $F(4.68, 585.35) = 4.20, MSE = 443.13, p = .001, \eta_p^2 = .033$ ($BF_{10} > 100$, decisive evidence, compared to null model), and a significant interaction between the two factors, $F(4.68, 585.35) = 4.33, MSE = 443.13, p < .001, \eta_p^2 = .034$ ($BF_{10} > 100$, decisive evidence, compared to two-main-effects model). Regarding the interaction, Holm-Bonferroni corrected pairwise comparisons suggested that physical tension mood ratings were significantly higher in the stress group compared to the control group at all time points 4 to 8, all $ps < .05$.

Subjective Ratings: Feeling of Control

Feeling of control was rated from 0 (“very low”) to 100 (“very high”). Means and standard deviations of the ratings are shown in Table S2. A 2 × 8 repeated-measures ANOVA with the factors of stress induction (stress vs. control) and time point of measurement (1 to 8), and number of participants per session as a covariate, showed a significant main effect of time point of measurement, $F(4.58, 572.15) = 4.36, MSE = 317.88, p = .001, \eta_p^2 = .034$ ($BF_{10} > 100$, decisive evidence,

compared to null model), and a significant interaction between the two factors, $F(4.58, 572.15) = 3.63, MSE = 317.88, p = .004, \eta_p^2 = .028$ ($BF_{10} = 56.44$, very strong evidence, compared to two-main-effects model). The main effect of stress induction was not significant, $F(1,125) = 3.12, MSE = 2552.05, p = .080$ ($BF_{01} = 1.26$, anecdotal evidence, compared to null model). Holm-Bonferroni corrected pairwise comparisons revealed that participants' feeling of control was significantly lower in the stress group than in the control group at time point 4, $p < .05$.

Subjective Ratings: Need for Contact

Need for social contact was rated from 0 ("no need at all") to 100 ("very high need"). Means and standard deviations of the ratings are shown in Table S2. A 2 × 8 repeated-measures ANOVA with the factors of stress induction (stress vs. control) and time point of measurement (1 to 8), and number of participants per session as a covariate, revealed a significant interaction between the two factors, $F(4.06, 507.98) = 4.20, MSE = 249.25, p = .002, \eta_p^2 = .032$ ($BF_{10} = 67.51$, very strong evidence, compared to two-main-effects model). Neither the main effect of stress induction, $F(1, 125) = 3.50, MSE = 4598.61, p = .064$ ($BF_{01} = 0.89$, anecdotal evidence, compared to null model), nor the main effect of time of measurement, $F(4.06, 507.98) < 1$, were significant, although Bayes analysis suggested decisive evidence for a main effect of time of measurement ($BF_{10} > 100$, compared to null model). Holm-Bonferroni corrected pairwise comparisons showed that need for contact was significantly stronger in the stress group than in the control group at time point 4, $p < .05$.

References

Dressendörfer, R. A., Kirschbaum, C., Rohde, W., Stahl, F., & Strasburger, C. J. (1992). Synthesis of a cortisol-biotin conjugate and evaluation as a tracer in an immunoassay for salivary cortisol measurement. *The Journal of Steroid Biochemistry and Molecular Biology*, *43*(7), 683–692. https://doi.org/10.1016/0960-0760(92)90294-S

Kirschbaum, C., Pirke, K.-M., & Hellhammer, D. H. (1993). The ‘Trier Social Stress Test’ – A tool for investigating psychobiological stress responses in a laboratory setting. *Neuropsychobiology*, *28*(1–2), 76–81. https://doi.org/10.1159/000119004

Lorentz, K., Gütschow, B., & Renner, F. (1999). *Evaluation of a direct α-amylase assay using 2-chloro-4-nitrophenyl-α-D-maltotrioside*. *37*(11–12), 1053–1062. https://doi.org/10.1515/CCLM.1999.154

von Dawans, B., Fischbacher, U., Kirschbaum, C., Fehr, E., & Heinrichs, M. (2012). The social dimension of stress reactivity: Acute stress increases prosocial behavior in humans. *Psychological Science*, *23*(6), 651–660. https://doi.org/10.1177/0956797611431576

von Dawans, B., Kirschbaum, C., & Heinrichs, M. (2011). The Trier Social Stress Test for Groups (TSST-G): A new research tool for controlled simultaneous social stress exposure in a group format. *Psychoneuroendocrinology*, *36*(4), 514–522. https://doi.org/10.1016/j.psyneuen.2010.08.004

Table S1

Table S1. Salivary cortisol and alpha-amylase levels as a function of group and time point of measurement. Means and standard deviations (in parentheses). Stress induction started following time point 3.

		Time Point of Measurement							
Variable	Group	1	2	3	4	5	6	7	8
Cortisol	**Stress**	4.87	5.14	4.70	4.24	5.49	6.87	7.16	5.42
(nmol/l)	(n=63)	(2.82)	(2.85)	(2.41)	(2.13)	(3.45)	(4.69)	(5.26)	(3.18)
	**Control**	4.24	4.48	4.23	4.11	3.90	3.93	3.87	3.30
	(n=64)	(2.04)	(2.59)	(2.40)	(2.34)	(2.24)	(2.55)	(2.57)	(1.97)
Alpha	**Stress**	166.57	141.13	151.28	194.90	269.34	226.90	168.39	173.77
Amylase	(n=62)	(122.71)	(98.91)	(101.31)	(136.36)	(199.19)	(171.90)	(112.92)	(114.60)
(U/l)	**Control**	171.28	135.46	139.82	158.95	183.59	170.77	137.14	153.51
	(n=64)	(113.90)	(98.00)	(89.33)	(114.10)	(124.94)	(112.10)	(92.70)	(106.15)

Table S2

Table S2. Subjective ratings as a function of group and time point of measurement.

Means and standard deviations (in parentheses). Stress induction started following time point 3.

		Time Point of Measurement							
Variable	Group	1	2	3	4	5	6	7	8
Subjective	**Stress**	29.69	24.22	40.47	62.50	60.16	53.75	40.62	22.66
Stress	(n=64)	(21.38)	(20.99)	(20.19)	(22.11)	(24.53)	(24.53)	(23.43)	(21.55)
	**Control**	24.06	20.31	35.00	35.78	35.62	39.37	32.34	14.06
	(n=64)	(22.94)	(20.08)	(21.53)	(23.89)	(22.25)	(24.94)	(23.08)	(15.81)
Mood	**Stress**	66.56	67.03	62.34	51.25	51.41	50.94	57.19	64.37
	(n=64)	(17.66)	(18.40)	(17.88)	(18.81)	(22.81)	(23.21)	(17.32)	(20.62)
	**Control**	63.91	68.75	64.84	62.03	63.91	62.97	62.66	71.41
	(n=64)	(15.80)	(16.18)	(15.43)	(16.25)	(16.00)	(15.60)	(15.66)	(15.72)
Physical	**Stress**	25.16	26.72	26.41	39.37	38.75	36.09	28.59	24.06
Wellbeing	(n=64)	(21.89)	(24.43)	(23.19)	(26.96)	(26.40)	(26.04)	(22.67)	(24.35)
	**Control**	26.41	21.72	22.34	27.34	28.59	31.72	28.12	20.78
	(n=64)	(21.70)	(17.69)	(16.11)	(19.78)	(21.00)	(22.79)	(19.26)	(16.36)
Physical	**Stress**	36.87	31.72	40.94	61.25	52.03	54.06	42.50	27.50
Tension	(n=64)	(23.43)	(26.40)	(22.66)	(22.99)	(28.01)	(26.83)	(24.56)	(24.62)
	**Control**	33.91	28.28	36.09	40.00	36.72	39.06	33.75	18.75
	(n=64)	(22.44)	(21.86)	(21.13)	(23.90)	(23.44)	(23.48)	(20.97)	(18.13)
Feeling of	**Stress**	65.62	67.03	60.31	43.28	50.62	50.00	57.34	71.72
Control	(n=64)	(21.07)	(21.87)	(17.81)	(19.44)	(24.42)	(21.60)	(21.40)	(23.06)
	**Control**	66.72	66.56	60.31	56.41	60.62	59.37	61.25	74.37
	(n=64)	(22.82)	(23.52)	(22.95)	(24.32)	(23.63)	(24.42)	(22.36)	(22.74)
Need for	**Stress**	31.56	27.97	25.31	40.94	36.09	34.37	29.22	26.72
Contact	(n=64)	(29.83)	(28.41)	(26.43)	(30.54)	(28.37)	(31.97)	(28.19)	(30.13)
	**Control**	29.84	25.16	24.06	27.34	26.25	25.16	22.66	19.69
	(n=64)	(26.34)	(23.02)	(24.02)	(25.28)	(24.33)	(23.84)	(22.06)	(22.46)
